# Intermittent enteral nutrition may increase gastrointestinal complications and mortality in critically ill patients

**DOI:** 10.3389/fnut.2025.1667836

**Published:** 2025-09-29

**Authors:** Panxin Hu, Haopeng Wu, Kai Zhang, Anan Li, Qiu Chen

**Affiliations:** ^1^Department of Emergency Medicine, The First People’s Hospital of Taizhou, Taizhou, China; ^2^Department of Critical Care Medicine, Second Affiliated Hospital, Zhejiang University School of Medicine, Hangzhou, China; ^3^Department of Cardiothoracic Surgery, The First People’s Hospital of Taizhou, Taizhou, China

**Keywords:** nutrition support, intermittent enteral nutrition, continuous enteral nutrition, critically ill, meta-analysis

## Abstract

**Background:**

Enteral nutrition (EN) is a cornerstone of nutritional support in critically ill patients. The optimal EN delivery strategy for critically ill patients remains controversial, with conflicting evidence regarding potential impacts on complications and clinical outcomes.

**Objectives:**

This meta-analysis aimed to compare the effects of intermittent enteral nutrition (IEN) versus continuous enteral nutrition (CEN) in critically ill patients.

**Methods:**

A comprehensive search of PubMed, Embase, Scopus, and the Cochrane Library was performed from inception to June 25, 2025. Randomized controlled trials (RCTs) comparing IEN and CEN in critically ill patients were included. Primary outcomes included gastrointestinal complications (diarrhea, abdominal distension, vomiting, constipation, gastric retention, and aspiration pneumonia), intensive care unit (ICU) mortality rate, length of ICU stay, and achievement of nutritional goal. Pooled relative risks (RRs) and mean differences (MDs) with 95% confidence intervals (CIs) were calculated using random-effects models.

**Results:**

Fifteen studies involving 1,406 patients were analyzed in this meta-analysis. In the overall critically ill population, IEN was associated with an increased incidence of diarrhea (RR 1.52, 95%CI 1.10 to 2.10, *I*^2^ = 16%) and abdominal distension (RR 2.38, 95%CI 1.17 to 4.83, *I*^2^ = 0%), higher ICU mortality (RR 1.39, 95%CI 1.02 to 1.89, *I*^2^ = 0%), and prolonged length of ICU stay (MD 0.81, 95%CI 0.18 to 1.45, *I*^2^ = 0%). Subgroup analysis further confirmed these findings in mechanically ventilated patients. In contrast, no significant differences in outcomes were observed between the two nutrition strategies in non-mechanically ventilated patients.

**Conclusion:**

This meta-analysis demonstrates that CEN appears superior to IEN among critically ill patients, particularly in those requiring mechanical ventilation. These results support for the preferential use of CEN in mechanically ventilated critically ill patients, while emphasizing the need for individualized nutritional management strategies that account for patient-specific factors and gastrointestinal tolerance.

**Systematic review registration:**

The study protocol was prospectively registered with the Open Science Framework (https://osf.io/krs8v).

## Introduction

Critical illness is characterized by a hypermetabolic state that significantly increases energy expenditure and protein catabolism, resulting in the rapid depletion of nutritional reserves and the onset of malnutrition without timely nutritional intervention (1). Malnutrition in critically ill patients is associated with heightened morbidity, prolonged mechanical ventilation, extended ICU stays, escalated healthcare costs, and an increased risk of mortality (2, 3). Recent guidelines continues to emphasize the critical role of optimal nutritional timing and delivery methods in improving patient outcomes, with emerging data suggesting that individualized approaches based on illness severity may be paramount (4, 5). Consequently, optimal nutritional support has emerged as a cornerstone of critical care medicine, with enteral nutrition (EN) being the preferred route when the gastrointestinal tract is functional (5, 6). However, the optimal method of EN delivery remains a subject of ongoing debate and investigation (7).

Two primary strategies have been employed in clinical practice: continuous and intermittent enteral feeding (8). Continuous enteral nutrition (CEN) involves the uninterrupted delivery of nutritional formula over 24 h, typically administered via feeding pumps at predetermined rates (9). This approach theoretically provides steady nutrient supply, maintains consistent serum glucose levels, and may reduce the risk of aspiration by minimizing gastric residual volumes. In contrast, intermittent enteral nutrition (IEN) involves the administration of nutritional formula in bolus doses at regular intervals, typically every 3 to 6 h, thereby mimicking the natural physiological pattern of food intake (8). Proponents of this method argue that intermittent feeding may better preserve normal gastrointestinal physiology, including gastric acid production, bile flow, and intestinal motility patterns (10).

Despite the theoretical advantages of each approach, clinical evidence regarding their comparative effectiveness remains inconsistent and fragmented. Several randomized controlled trials (RCTs) have investigated the differences between continuous and intermittent feeding methods, examining various outcomes including gastrointestinal tolerance, nutritional adequacy, and clinical outcomes. However, these individual studies have yielded conflicting results, with some favoring continuous delivery (11, 12), while others suggest benefits of intermittent approaches (13–15).

In light of the clinical significance of optimizing nutritional support for critically ill patients and the conflicting findings from individual studies, a comprehensive meta-analysis of the available RCTs is imperative. Therefore, we undertook this systematic review and meta-analysis to evaluate and compare the effectiveness and safety of IEN versus CEN strategies in critically ill patients. Our primary objectives were to evaluate the comparative effects of these two approaches on gastrointestinal tolerance, nutritional adequacy, and clinical outcomes. By synthesizing the evidence from RCTs, this meta-analysis seeks to provide clinicians with evidence-based guidance in selecting the most suitable enteral nutrition delivery strategy for critically ill patients. Ultimately, this will contribute to enhanced patient outcomes and optimized utilization of resources within the intensive care environment.

## Methods

### Study design and registration

This systematic review and meta-analysis was conducted in accordance with the Preferred Reporting Items for Systematic Reviews and Meta-Analyses (PRISMA) guidelines (16), with the PRISMA checklist provided in Supplementary material 1. The study protocol was prospectively registered with the Open Science Framework.^1^

### Search strategy

A comprehensive literature search was performed to identify relevant randomized controlled trials comparing IEN versus CEN strategies in critically ill patients. The following electronic databases were systematically searched from inception to June 25th, 2025: PubMed, Embase, Scopus, and the Cochrane Library. The search strategies are documented in Supplementary material 2.

### Inclusion criteria

Studies meeting the following inclusion criteria were included: (1) Population: Adult critically ill patients (≥18 years) requiring enteral nutritional support; (2) Intervention: IEN delivery (bolus or cyclic feeding); (3) Comparison: CEN delivery; (4) Outcomes: Primary outcomes were gastrointestinal complications (diarrhea, abdominal distension, vomiting, constipation, gastric retention, and aspiration pneumonia). Secondary outcomes assessed were ICU mortality, length of ICU stay, and nutritional goal attainment. The definitions of outcomes were consistent with those used in the included trials; (5) Study design: Randomized trials.

### Exclusion criteria

Studies were excluded if they: (1) Enrolled pediatric populations (<18 years); (2) Involved patients receiving parenteral nutrition as the primary intervention; (3) Compared different types of enteral nutrition formulas rather than delivery methods; (4) Were non-randomized studies, case reports, case series, or review articles; (5) Lacked sufficient data for meta-analysis; (6) Included patients with specific gastrointestinal conditions that would preclude standard enteral nutrition protocols.

### Data extraction

Two independent investigators conducted initial screening of titles and abstracts from all retrieved literature to assess potential eligibility. Subsequently, full-text manuscripts of candidate studies underwent independent evaluation by the same research team. Discrepancies were resolved through collaborative discussion or arbitration by a third investigator when consensus could not be reached.

Data abstraction was performed independently by two reviewers employing a standardized extraction template. The following parameters were systematically retrieved from each qualifying study: study attributes (principal author, publication year, research methodology), participant demographics (sample size, age distribution, gender composition, body mass index, disease severity metrics), and intervention specifications (nutritional delivery approach, treatment duration, and feeding regimens).

### Quality assessment

Two independent reviewers assessed the risk of bias for each included randomized controlled trial using the Cochrane RoB 2.0 tool (17). Assessments were conducted at the outcome level, evaluating five domains: (1) randomization process, (2) deviations from intended interventions, (3) missing outcome data, (4) outcome measurement, and (5) selection of reported results. Signaling questions within each domain guided judgments categorized as low risk, some concerns, or high risk. The overall risk of bias for each study was determined by integrating domain-level judgments, adhering to the RoB 2.0 decision algorithms. Discrepancies were resolved through consensus or third-reviewer consultation.

### Statistical synthesis and analysis

Statistical analyses were performed using R statistical software version 4.0 with the “meta” and “robvis” packages. For dichotomous outcomes, pooled relative risks (RR) with 95% confidence intervals (CI) were calculated using the Mantel–Haenszel method. For continuous outcomes, mean differences with 95% CI were calculated using the inverse variance method. Statistical heterogeneity was evaluated using the chi-square test and *I*^2^ statistic (18), where *I*^2^ values of 25, 50, and 75% denoted low, moderate, and high heterogeneity, respectively. A random-effects model was used for all analyses to account for potential clinical and methodological heterogeneity between studies. Publication bias was assessed using funnel plot visual inspection and statistical tests including Egger’s regression test when at least 8 studies were available for a specific outcome (19).

A leave-one-out sensitivity analysis was conducted by iteratively excluding each included study and recalculating the pooled effect estimates. This approach assessed the influence of individual studies on overall results and tested the robustness of conclusions. To evaluate whether mechanical ventilation (MV) status modifies the effects of different EN strategies, we performed a predefined subgroup analysis. Patients were stratified into two subgroups: MV subgroup and non-MV subgroup. Given the wide range of APACHE II scores across included studies, we performed an additional predefined subgroup analysis stratified by illness severity. Studies were categorized based on mean APACHE II scores: moderate severity (APACHE II <20) and high severity (APACHE II ≥20).

## Results

### Study characteristics

**Figure 1:**
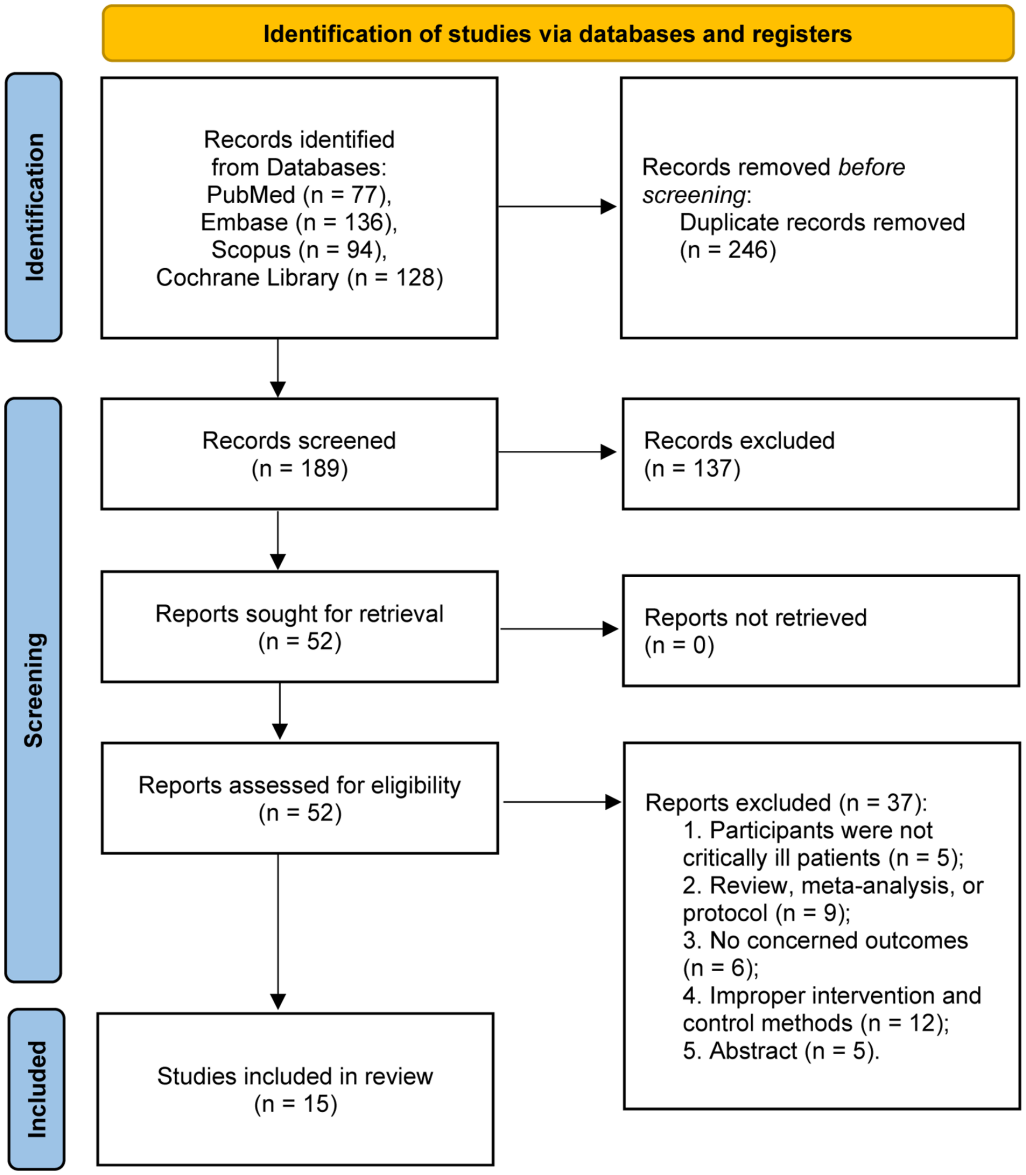
PRISMA 2020 flow diagram for this meta-analysis.

The systematic literature search identified 435 potentially relevant articles across all databases. Following duplicate removal, 189 unique records underwent title and abstract screening. Subsequently, 52 full-text manuscripts were evaluated for eligibility. Upon comprehensive assessment, 15 randomized controlled trials satisfied the inclusion criteria and were incorporated into the final meta-analysis (11–15, 20–29). The study selection procedure is depicted in the PRISMA flow diagram (Figure 1).

A total of 1,406 patients were analyzed, with 708 patients receiving IEN and 698 patients receiving CEN during the study periods. Patient populations included general ICU patients (*n* = 10), trauma patients (*n* = 3), septic patients (*n* = 1), and patients with hemorrhagic stroke (*n* = 1), 10 studies reported the illness severity scores, with the average APACHE II scores ranging from 12 to 29, indicating moderate to high illness severity.

Regarding intervention characteristics, IEN protocols varied across studies, with feeding intervals ranging from 3 to 8 h (most commonly every 4 h). CEN was delivered over 24 h in 14 studies, the remaining 2 studies (13, 29) administrated CEN for 18 h per day. The duration of nutritional intervention ranged from 3 to 14 days, the majority of included studies compared the two feeding regimens in a 7-day study period. Study characteristics are detailed in Table 1.

**Table 1:** Characteristics of studies included in the meta-analysis.

Study and year	Design	Number of patients (intermittent/continuous)	Population	Characteristics (intermittent/continuous)	Intervention		Study period
					Intermittent	Continuous	
Hrdy et al., 2025 (13)	Unblinded, single-center	146/148	Patients ≥18 years, required MV for ≥72 h in ICU	Age: 62.9/64.4; Male (%): 71/74; BMI: 29.0/29.0; APACHE II: 28/29	0.5 to 1 h for 6 times daily, infusion rate was 160 to 400 mL/h	18 h daily, initial infusion rate was 25 mL/h	5 days
Yao et al., 2025 (28)	Unblinded, single-center	65/69	Adult patients ≥18 years in ICU	Age: 64/65; Male (%): 69/80; APACHE II: 16/16	Within 2 h for 3 times daily, infusion rate was decided by researchers	24 h daily, infusion rate was decided by researchers	7 days
Panwar et al., 2024 (27)	Unblinded, multicenter	61/59	Adult ICU patients had gastric feeding tube, received invasive MV	Age: 65.1/64.8; Male (%): 67/66; BMI: 27.9/27.2; APACHE II: 22/23	0.5 to 1 h for 3 times daily, initial rate was 150 mL/h	24 h daily, according to current standard practice	14 days
Wilkinson et al., 2023 (26)	Single-blinded, multicenter	40/35	Patients ≥18 years, required MV for ≥48 h in ICU	Age: 55/61; Male (%): 28/26; APACHE II: 21/20	3 to 5 min for 6 times daily, infusion rate was 60 to 80 mL a time	24 h daily, infusion rate was decided by researchers	10 days
Lee et al., 2022 (11)	Unblinded, single-center	49/50	Patients ≥20 years, required MV	Age: 66.2/67.5; Male (%): 67.3/66.0; BMI: 22.0/23.3; APACHE II: 27.7/28.6	1 h for 4 times daily, initial rate was 150 mL/h	24 h daily, initial rate was 25 mL/h	7 days
Ren et al., 2021 (25)	Single-blinded, single-center	32/30	Patients admitted to ICU and fed through gastric tubes	Age: 66/55; Male (%): 53/63; BMI: 24/23; APACHE II: 19/16	2 h for 3 times daily, infusion rate was decided by researchers	24 h daily, infusion rate was decided by researchers	7 days
Zhu et al., 2020 (12)	Single-blinded, single-center	40/38	Patients ≥18 years, with hemorrhagic stroke, required MV	Age: 59.9/59.6; Male: 55.3/47.5; BMI: 24.6/24.1	0.5 to 1 h for 4 times daily, infusion rate was decided by researchers	24 h daily, maximum rate was 100 mL/h	7 days
McNelly et al., 2020 (14)	Single-blinded, multicenter	62/59	Patients ≥18 years, required MV for ≥48 h	Age: 55.2/60.3; Male: 66.1/67.8; APACHE II: 23.1/20.2	6 times daily, according to the individual nutritional targets	24 h daily, according to local feeding protocol	10 days
Nasiri et al., 2017 (24)	Triple-blinded, single-center	20/20	Patients aged between 18 to 65 years, admitted to ICU with sepsis	Age: 48.3/53.0; Male: 26.5/35.3	15 to 20 min for 6 times daily, infusion rate was 50 to 200 mL a time	24 h daily, infusion rate was decided by researchers	3 days
Kadamani et al., 2014 (23)	Unblinded, single-center	15/15	Patients aged between 20 to 80 years, received MV and EN for ≥72 h	Age: 61.6/64.7; Male: 66.7/60.0; BMI: 23.3/23.1; APACHE II: 16.0/20.3	10 to 15 min every 4 to 6 h, infusion rate was decided by researchers	24 h daily, infusion rate was decided by researchers	3 days
Maurya et al., 2011 (29)	Unblinded, single-center	20/20	Adult male patients age of 20 to 60 years with history of head injury requiring MV	Age: 40.2/40.7; Male: 100/100; BMI: 22.0/20.6; SOFA: 2.8/2.7	6 times daily, infusion rate was 220 mL a time	18 h daily, infusion rate was decided by researchers	NR
MacLeod et al., 2007 (15)	Unblinded, single-center	79/81	Patients ≥18 years, admitted to trauma ICU, required MV ≥48 h	Age: 44.6/48.4; Male: 67.1/74.1; APACHE II: 12/14	100 mL of formula within 30 to 60 min for 6 times daily	24 h daily, initial rate was 20 mL/h	7 days
Chen et al., 2006 (22)	Unblinded, single-center	56/51	Patients ≥20 years, required MV in ICU	Male: 76.8/76.5; APACHE II: 22.6/22.6	125 mL of formula for 6 times daily	24 h daily, initial rate was 25 mL/h	7 days
Serpa et al., 2003 (21)	Unblinded, single-center	14/14	Adult patients ≥18 years in ICU	Age: 64.9/69.6; Male: 50.0/64.3	1 h for 6 times daily, infusion rate was decided by researchers	24 h daily, infusion rate was decided by medical indications	3 days
Steevens et al., 2002 (20)	Unblinded, single-center	9/9	Multiple trauma patients age of 18 to 70 years admitted to ICU	Age: 35.9/37.3; Male: 55.6/77.8; BMI: 27.5/25.4; ISS: 34/34	125 mL within 15 min for 6 times daily	24 h daily, initial rate was 25 mL/h	7 days

BMI, body mass index; APACHE II, Acute Physiology and Chronic Health Evaluation II; ISS, Injury Severity Score; SOFA, Sequential Organ Failure Assessment Score; ICU, intensive care unit; GCS, Glasgow Coma Scale; EN, enteral nutrition.

### Quality assessment

**Figure 2:**
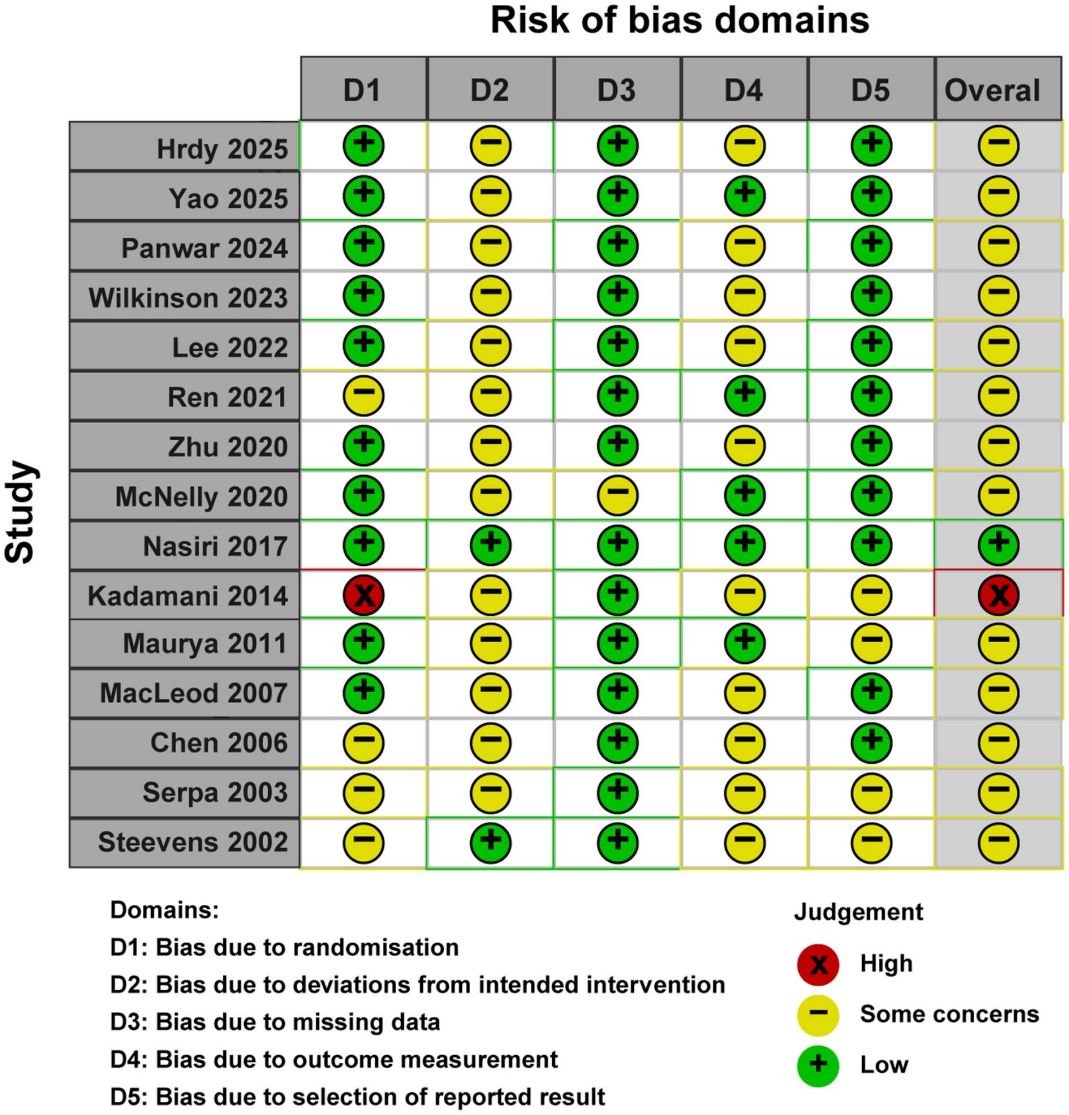
Risk of bias 2 of all included studies.

The methodological quality assessment using the Cochrane Risk of Bias tool (RoB 2.0) indicated that one study exhibited an overall low risk of bias, 13 studies raised some concerns, and one study was deemed to have a high risk of bias. The most prevalent sources of bias pertained to deviations from intended interventions due to the inherent nature of the intervention, and to outcome measurement. A detailed risk of bias evaluation is depicted in Figure 2.

Furthermore, publication bias was assessed using Egger’s test and a funnel plot, which revealed no significant evidence of publication bias (Egger’s test, *p* > 0.05; Supplementary material 3).

### Outcomes

**Figure 3:**
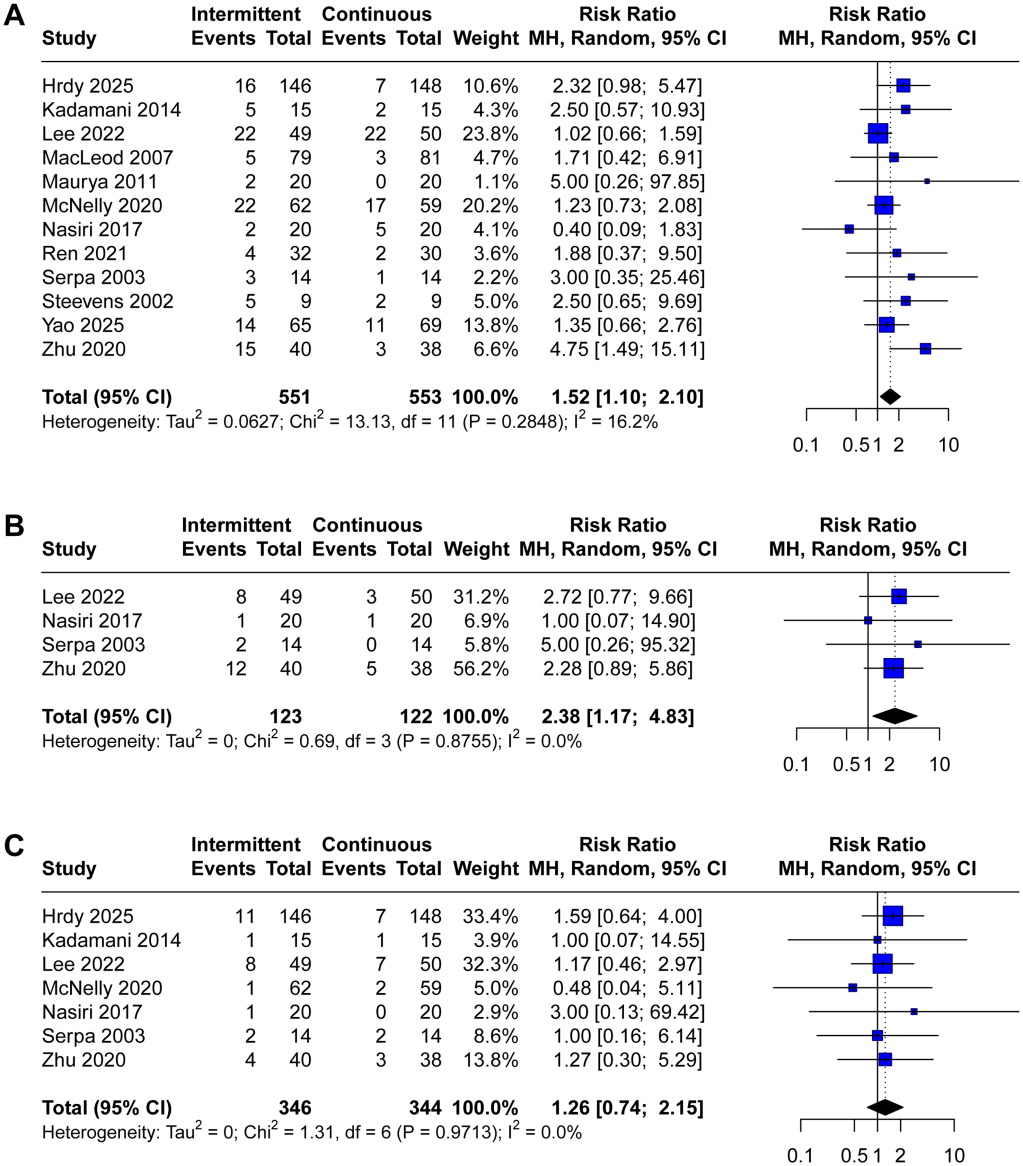
Forest plot comparing the effect of IEN versus CEN on **(A)** diarrhea, **(B)** abdominal distension, **(C)** vomiting.

**Figure 4:**
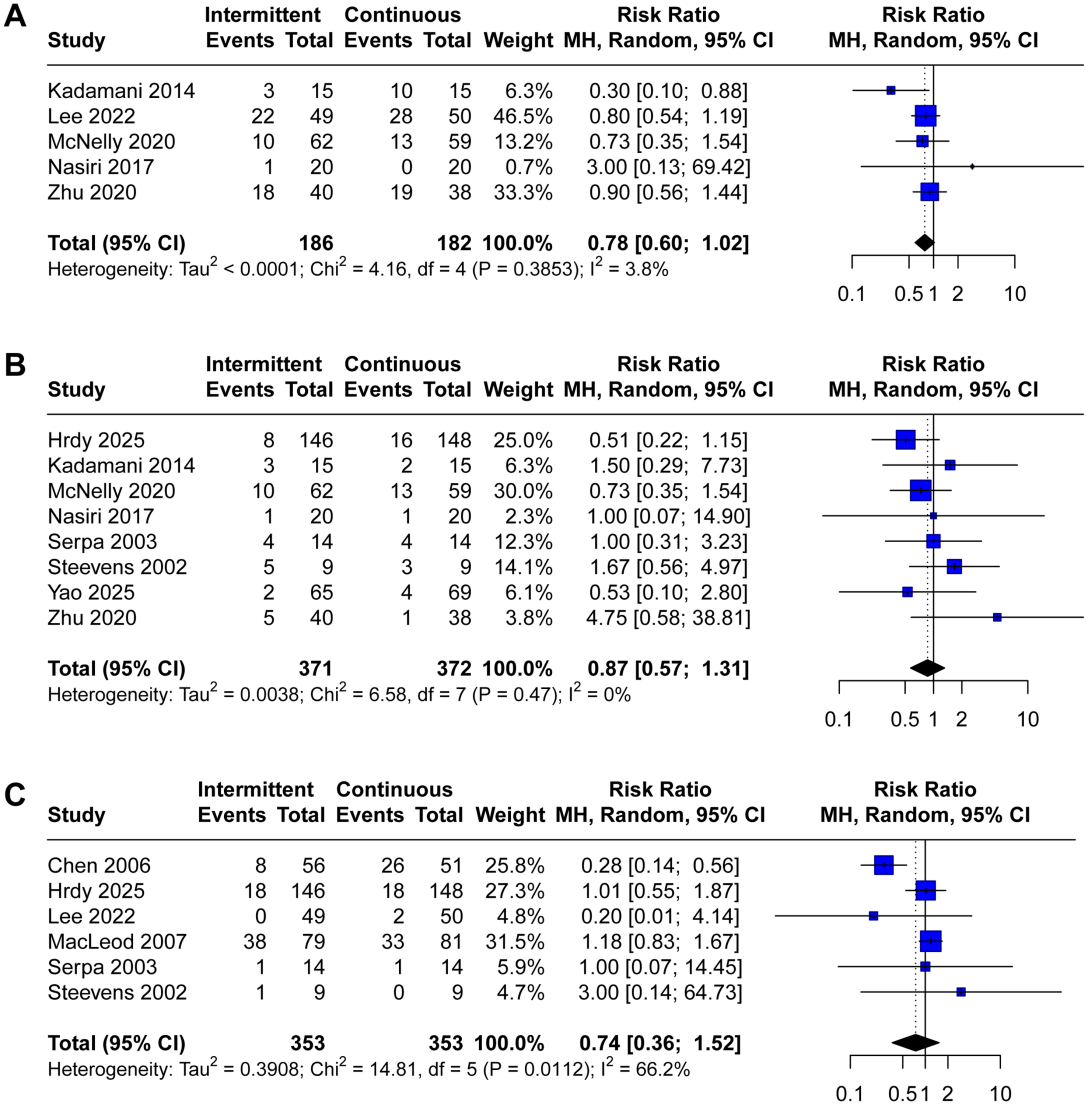
Forest plot comparing the effect of IEN versus CEN on **(A)** constipation, **(B)** gastric retention, **(C)** aspiration pneumonia.

A total of 12 trials reported the incidence of gastrointestinal complications, including diarrhea (*n* = 12), abdominal distension (*n* = 4), vomiting (*n* = 7), constipation (*n* = 5), gastric retention (*n* = 8), and aspiration pneumonia (*n* = 6). The meta-analysis demonstrated that IEN was associated with a higher incidence of diarrhea (RR 1.52, 95% CI 1.10 to 2.10, *I*^2^ = 16%, Figure 3A) and abdominal distension (RR 2.38, 95% CI 1.17 to 4.83, *I*^2^ = 0%, Figure 3B). No significant differences were identified between IEN and CEN for other gastrointestinal complications (vomiting: RR 1.26, 95% CI 0.74 to 2.15, *I*^2^ = 0%, Figure 3C; constipation: RR 0.78, 95% CI 0.60 to 1.02, *I*^2^ = 4%, Figure 4A; gastric retention: RR 0.87, 95% CI 0.57 to 1.31, *I*^2^ = 0%, Figure 4B; aspiration pneumonia: RR 0.74, 95% CI 0.36 to 1.52, *I*^2^ = 66%, Figure 4C).

**Figure 5:**
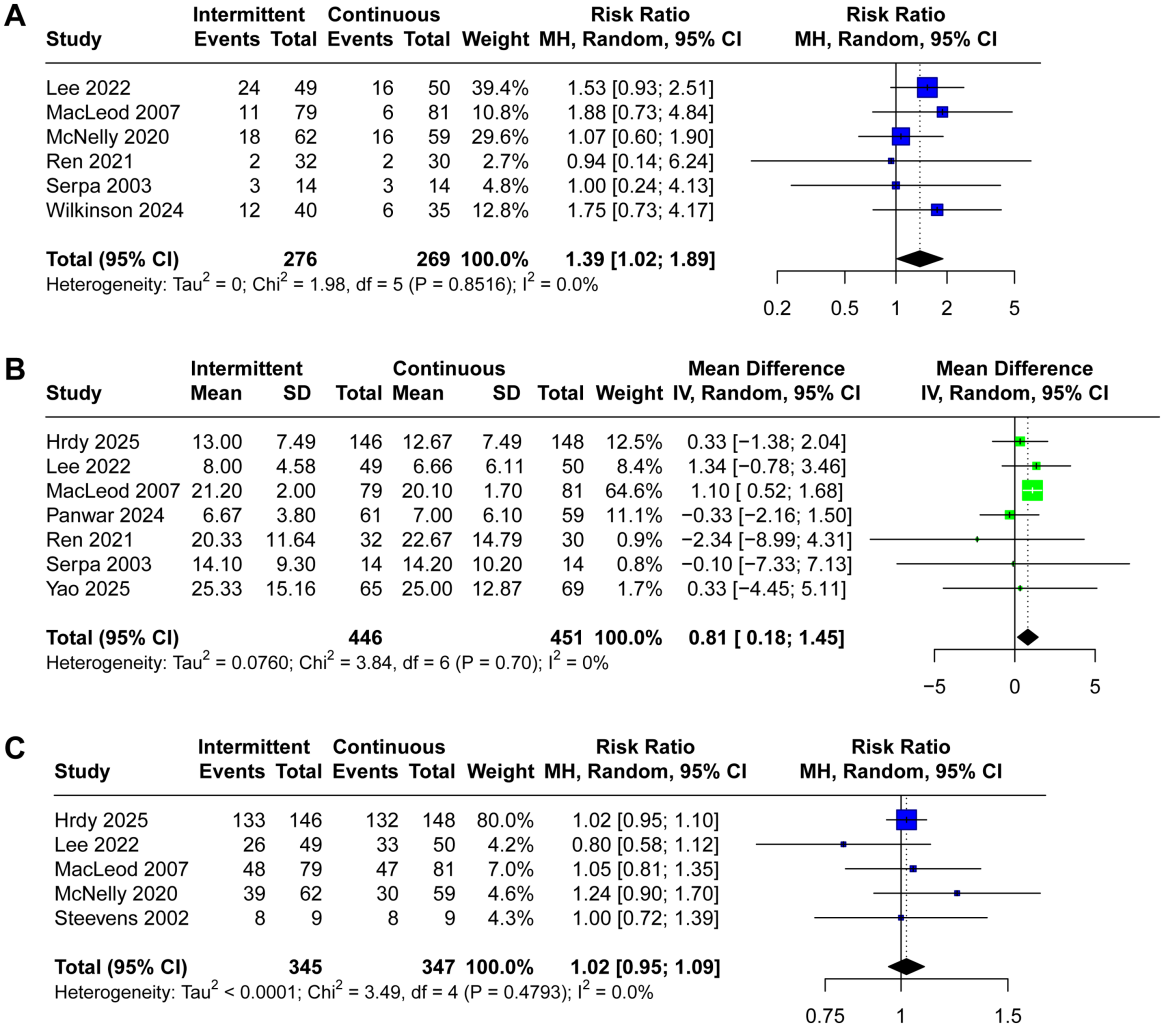
Forest plot comparing the effect of IEN versus CEN on **(A)** ICU mortality rate, **(B)** length of ICU stay, **(C)** achievement of nutritional goal.

ICU mortality, length of stay in ICU, and achievement of nutritional goal were reported in six, seven, and five trials, respectively. Critically ill patients receiving IEN had higher ICU mortality (RR 1.39, 95% CI 1.02 to 1.89, *I*^2^ = 0%, Figure 5A) and longer length of stay in ICU (MD 0.81, 95% CI 0.18 to 1.45, *I*^2^ = 0%, Figure 5B). No significant difference was observed between the two groups regarding achievement of nutritional goal (RR 1.02, 95% CI 0.95 to 1.09, *I*^2^ = 1%, Figure 5C).

### Sensitivity and subgroup analyses

When we evaluated the influence of each individual trial on the pooled estimates through sensitivity analysis, we observed that the exclusion of certain studies could alter the findings. Specifically, the incidence of abdominal distension lost statistical significance upon excluding the trials by Lee et al. (11), and Zhu et al. (12) (Supplementary material 3). Similarly, omitting the studies by Lee et al. (11), MacLeod et al. (15), and Wilkinson et al. (26) affected the outcomes for ICU mortality and ICU length of stay (Supplementary material 3). These sensitivity analyses indicate that the results lack sufficient robustness.

In the subgroup analysis of mechanically ventilated patients (Supplementary material 3), IEN was associated with higher incidence of diarrhea (RR 1.69, 95% CI 1.07 to 2.68, *I*^2^ = 35%) and abdominal distension (RR 2.43, 95% CI 1.14 to 5.18, *I*^2^ = 0%), higher ICU mortality (RR 1.42, 95% CI 1.03 to 1.97, *I*^2^ = 0%), and longer length of stay in ICU (MD 0.88, 95% CI 0.28 to 1.48, *I*^2^ = 0%). However, in contrast to the mechanically ventilated subgroup, the analysis of non-mechanically ventilated patients revealed no statistically significant differences between IEN and CEN strategies for any of the evaluated outcomes. Neither gastrointestinal complications (including diarrhea, and abdominal distension) nor clinical outcomes (ICU mortality and length of stay in ICU) showed significant differences between the two feeding strategies in this patient population (Supplementary material 3).

Furthermore, the higher severity subgroup (APACHE II ≥20) demonstrated IEN was associated with higher incidence of diarrhea (RR 1.69, 95% CI 1.00 to 2.84, *I*^2^ = 53%) and abdominal distension (RR 2.43, 95% CI 1.14 to 5.18, *I*^2^ = 0%), higher ICU mortality (RR 1.37, 95% CI 0.97 to 1.94, *I*^2^ = 0%). In the lower severity subgroup (APACHE II <20), longer length of stay in ICU (MD 1.06, 95% CI 0.49 to 1.62, *I*^2^ = 0%) was observed with IEN (Supplementary material 3).

## Discussion

### Principal findings

This meta-analysis of 15 RCTs provides comprehensive evidence comparing IEN versus CEN in critically ill patients. Our findings reveal that IEN is associated with increased rates of diarrhea and abdominal distension, prolonged ICU length of stay, and higher ICU mortality compared to CEN. Notably, these effects were predominantly observed in mechanically ventilated patients, with no significant differences detected in non-mechanically ventilated critically ill patients.

### Clinical implications and mechanistic considerations

Gastrointestinal complications are common in critically ill patients receiving EN. Research has demonstrated that over 40% of critically ill patients experience various gastrointestinal complications during EN support (30). In this comprehensive and up-to-date meta-analysis, the increased incidence of diarrhea and abdominal distension associated with IEN may be attributed to the rapid and bolus-like delivery of nutrients, which may exceed the gastrointestinal tolerance capacity in critically ill patients. Intermittent feeding may overwhelm the digestive system’s capacity for nutrient absorption, leading to osmotic diarrhea and malabsorption (31). The intermittent delivery of large volumes may also trigger rapid gastric emptying and accelerated intestinal transit, contributing to loose stools (32). Conversely, continuous feeding allows for steady-state nutrient absorption and may better preserve normal gastrointestinal physiology. These findings indicate that CEN may be a preferred EN strategy for critically ill patients to reduce the incidence of gastrointestinal complications, which is consistent with recommendations from American Society for Parenteral and Enteral Nutrition guidelines (33).

The association between IEN and increased ICU mortality warrants careful consideration. While the underlying mechanism remains unclear, several hypotheses merit discussion. First, the increased gastrointestinal complications associated with IEN may compromise nutritional adequacy, leading to protein-energy malnutrition and impaired immune function (34). Second, the higher incidence of diarrhea may predispose patients to electrolyte imbalances, dehydration, and secondary infections (35). The prolonged length of ICU stay observed with intermittent feeding may reflect the cumulative impact of increased gastrointestinal complications. The time required to manage feeding-related complications may delay patient recovery and discharge readiness. A meta-analysis of 26 studies demonstrated that gastrointestinal complications were significantly associated with all-cause hospital mortality (odds ratio 1.62, 95% CI 1.14 to 2.30) and prolonged length of hospital stay (MD 5.31, 95% CI 2.96 to 7.67) (30).

The subgroup analysis suggests that the effects of IEN versus CEN are modified by ventilator support status, with mechanically ventilated patients showing more pronounced differences in outcomes between the two feeding strategies. Importantly, the observed APACHE II score range may contribute to relevance bias. Patients with lower scores may tolerate IEN better, whereas those with higher scores are critically unstable, more prone to gastrointestinal dysfunction, and thus less tolerant to IEN. This variability likely explains why significant differences emerged in ventilated patients (who often represent the higher-severity group), but not in non-ventilated patients. Future studies should stratify analyses by illness severity (e.g., APACHE II) to mitigate this bias.

The differential effects observed between mechanically ventilated and non-mechanically ventilated patients provide important insights into the pathophysiology of feeding tolerance. Mechanically ventilated patients often have compromised gastrointestinal function due to sedation, neuromuscular blocking agents, and altered autonomic regulation (36, 37). These factors may impair gastric emptying and intestinal motility, making these patients more susceptible to feeding intolerance with intermittent bolus delivery. These findings underscore the need for individualized approaches based on patient-specific factors such as gastrointestinal function, sedation level, and ventilator support status.

### Comparison with existing literature

Our findings differ somewhat from prior research: Ma et al. (38) analyzed 14 RCTs published prior to 2020 and found that intermittent feeding was associated with an increased incidence of feeding intolerance, but revealed no significant differences in other clinical parameters. Apart from the relatively older publication years of the meta-analysis by Ma et al. (38), a majority of the included RCTs were conducted in China, the generalizability of their findings to international contexts may be limited. Subsequently, Heffernan et al. (39) conducted a meta-analysis including 14 English-language RCTs, they found no significant difference in mortality, diarrhea, increased gastric residuals, pneumonia, and bacterial colonization. Another meta-analysis by Qu et al. (40) indicated that intermittent enteral feeding was associated with an increased incidence of diarrhea and abdominal distension, as well as a longer ICU length of stay, but reduced constipation rates. However, two of the RCTs (41, 42) included by Heffernan et al. (39) and Qu et al. (40) employed an 18-h daily infusion protocol to represent intermittent enteral feeding. The relatively minimal difference between this intervention and continuous enteral feeding (administered over 24 h per day) may have contributed to the lack of significant differences observed in some outcomes.

A significant contribution of our study is the demonstration that IEN is associated with increased ICU mortality and prolonged ICU length of stay compared to CEN. This finding represents a departure from previous meta-analyses that primarily focused on feeding tolerance and gastrointestinal complications, with limited attention to clinical outcomes. Furthermore, our subgroup analysis reveals that the adverse effects of IEN are predominantly observed in mechanically ventilated patients, while showing no significant impact in non-mechanically ventilated patients. Recently, Panwar et al. (43) performed a meta-analysis to compare continuous feeding versus intermittent feeding among mechanically ventilated patients. The meta-analysis of 8 RCTs did not detect any significant differences in important clinical outcomes (including mortality, ICU length of stay, gut intolerance, and pneumonia) between the two groups (43). By incorporating the most recent RCTs, our meta-analysis provides important clinical nuance that has not been adequately addressed in previous researches.

### Clinical practice recommendations

Based on our findings, CEN should be considered the preferred strategy for critically ill patients, particularly those requiring mechanical ventilation. The evidence suggests that CEN is associated with better clinical outcomes, including reduced mortality, shorter ICU length of stay, and improved gastrointestinal tolerance. For patients who develop constipation with CEN, targeted interventions such as prokinetic agents or fiber supplementation may be more appropriate than switching to IEN, given the associated risks of the latter strategy. Given the observed association between IEN and worse clinical outcomes, our findings have important implications for nutritional support strategies in critically ill patients. Continuous EN should remain the preferred approach in routine clinical practice, particularly for patients at higher risk of gastrointestinal intolerance or those requiring mechanical ventilation.

Future large-scale RCTs are warranted to explore whether certain subgroups may benefit from intermittent EN, such as patients with preserved gastrointestinal motility or those in the recovery phase of critical illness. Moreover, further research should also investigate long-term outcomes to determine whether the observed differences in ICU mortality translate to differences in hospital mortality or long-term survival. Furthermore, economic analyses comparing the cost-effectiveness of different feeding strategies, considering both direct medical costs and length of stay implications, would provide valuable information for healthcare decision-making.

Clinicians should carefully consider the risk–benefit profile when selecting feeding strategies, particularly considering patient-specific factors such as baseline gastrointestinal function, severity of illness, and individual tolerance patterns. Recent guidelines have markedly shifted from standardized protocols toward a more personalized approach, emphasizing the necessity of tailoring nutritional support (4, 5). The development of evidence-based guidelines incorporating these findings could standardize practice and improve patient outcomes.

### Limitations

Several limitations should be acknowledged when interpreting these results. First, the inherent differences between the two feeding strategies rendered blinding of participants and investigators impractical. As a result, the majority of included RCTs in our meta-analysis were characterized by moderate-to-high risk of bias. Second, the definition of “intermittent” feeding varied across studies, with different bolus volumes, frequencies, and administration methods potentially influencing outcomes. Similarly, the assessment of gastrointestinal tolerance varied between studies, with some relying on subjective measures while others used more objective criteria. The follow-up duration and timing of outcome assessment also varied across studies, potentially affecting the comparability of results. Third, this meta-analysis included several RCTs conducted primarily in China, which may limit the generalizability of our findings to other populations due to potential differences in dietary habits, healthcare systems, and genetic factors. Future studies should prioritize diverse geographic settings to validate these results globally. Additionally, many studies did not adequately report feeding adequacy or achievement of nutritional goals, which could be important mediating factors in the observed clinical outcomes.

## Conclusion

This meta-analysis provides robust evidence that CEN is associated with superior clinical outcomes compared to IEN in critically ill patients. The increased mortality, prolonged ICU stay, and higher rates of diarrhea and abdominal distension associated with IEN support the preferential use of continuous feeding strategies in this population. These findings are particularly relevant for mechanically ventilated patients, who appear to be at highest risk for complications with intermittent feeding. Healthcare providers should consider these evidence-based recommendations when developing nutritional support protocols for critically ill patients, while recognizing the need for individualized approaches based on patient-specific factors and clinical circumstances.

## Data Availability

The original contributions presented in the study are included in the article/Supplementary material, further inquiries can be directed to the corresponding author.

## Footnotes

^1^ https://osf.io/krs8v
