# Supplementary material for: Intermittent enteral nutrition may increase gastrointestinal complications and mortality in critically ill patients
Source: Front Nutr. 2025 Sep 29;12:1667836. doi: 10.3389/fnut.2025.1667836 (PMC12515623; doi:10.3389/fnut.2025.1667836)
Supplement: Supplementary file 3 [file Table_3.DOCX]

**Supplementary Material 3:** Publication bias assessment by funnel plot and Egger’s test, sensitivity and subgroup analyses.


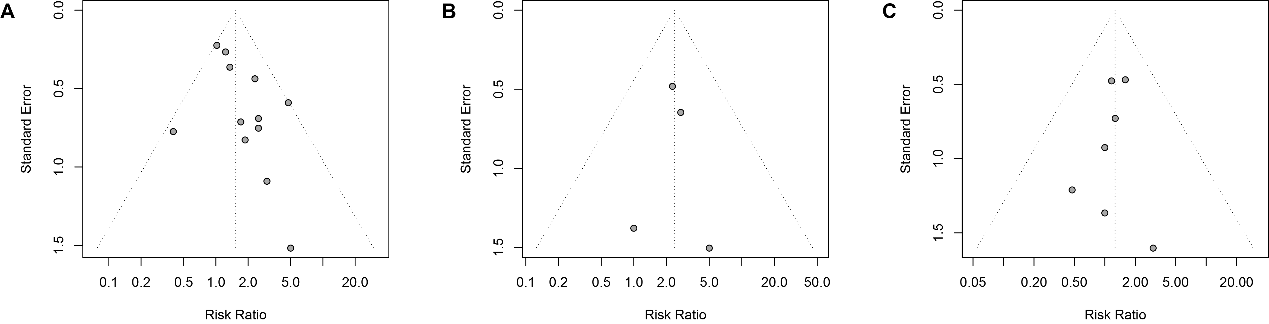


Figure 2: Funnel plot for (A) diarrhea, Egger’s test P=0.0681, (B) abdominal distension, Egger’s test P=0.9686, (C) vomiting, Egger’s test P=0.5517


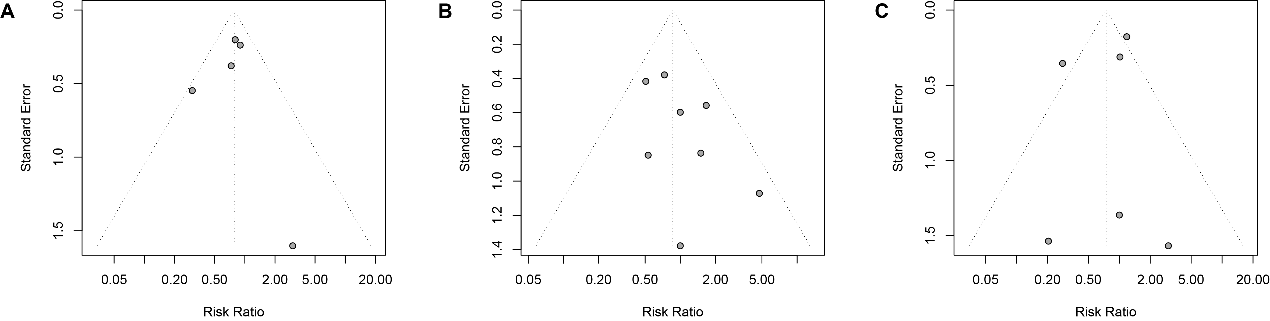


Figure 3: Funnel plot for (A) constipation, Egger’s test P=0.7929, (B) gastric retention, Egger’s test P=0.1614, (C) aspiration pneumonia, Egger’s test P=0.5976


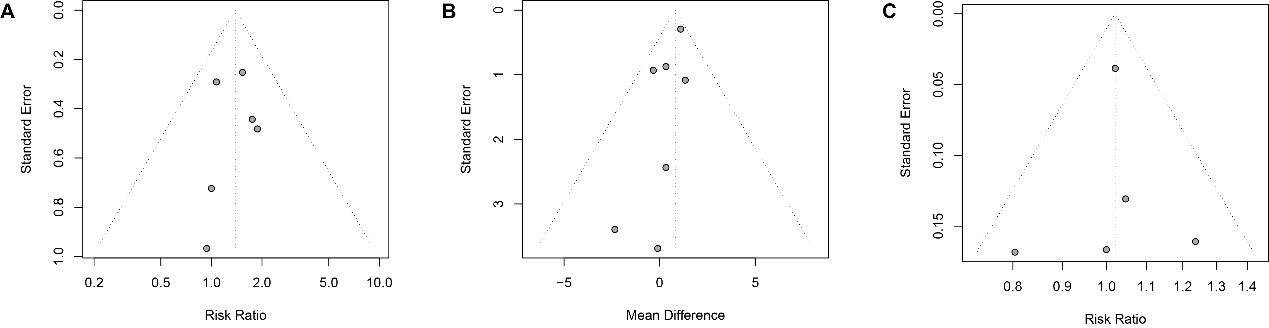


Figure 4: Funnel plot for (A) ICU mortality, Egger’s test P=0.8067, (B) length of stay in ICU, Egger’s test P=0.0987, (C) achievement of nutritional goal, Egger’s test P=0.9263


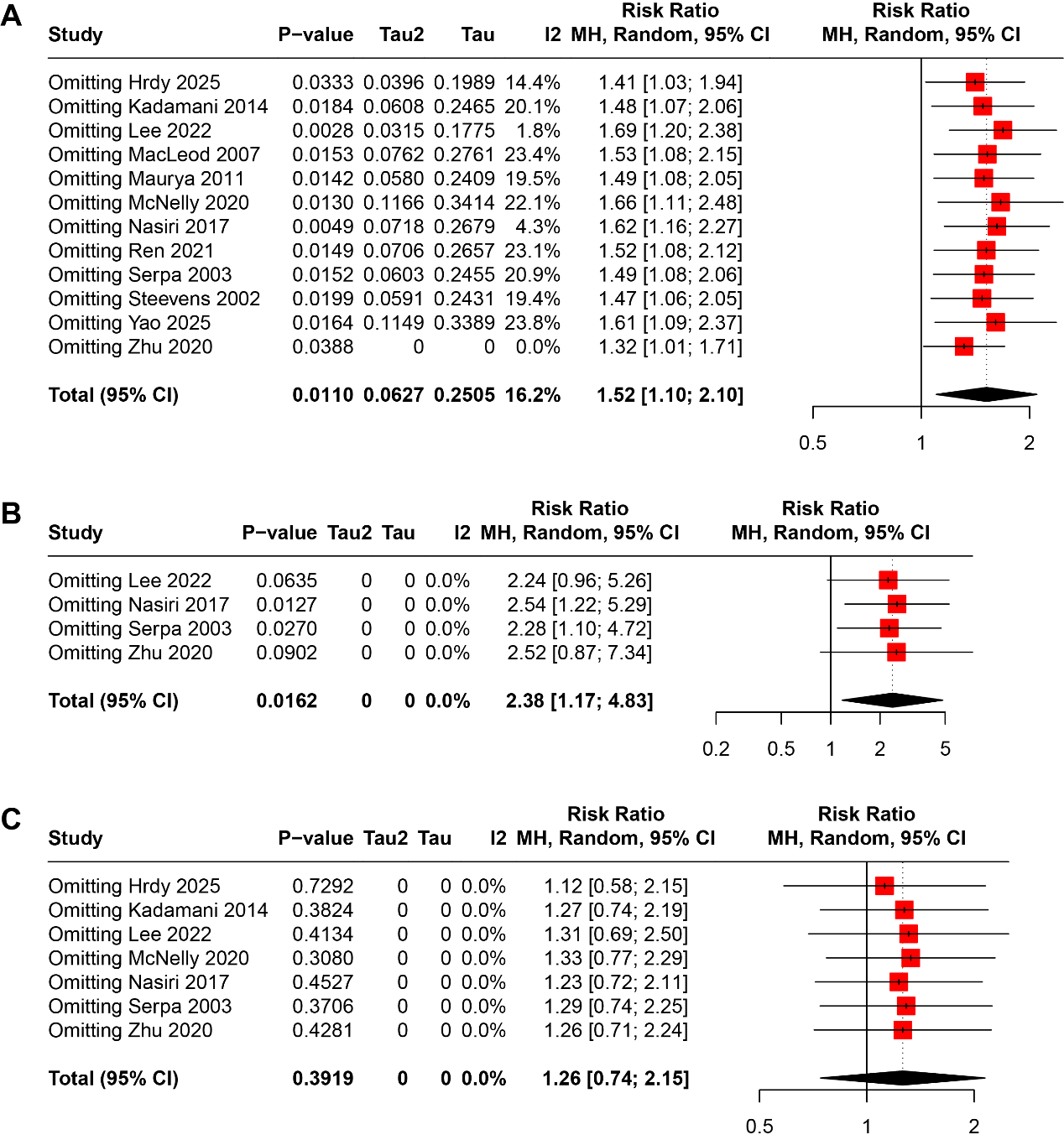


Figure 5: Sensitivity analysis for (A) diarrhea, (B) abdominal distension, (C) vomiting


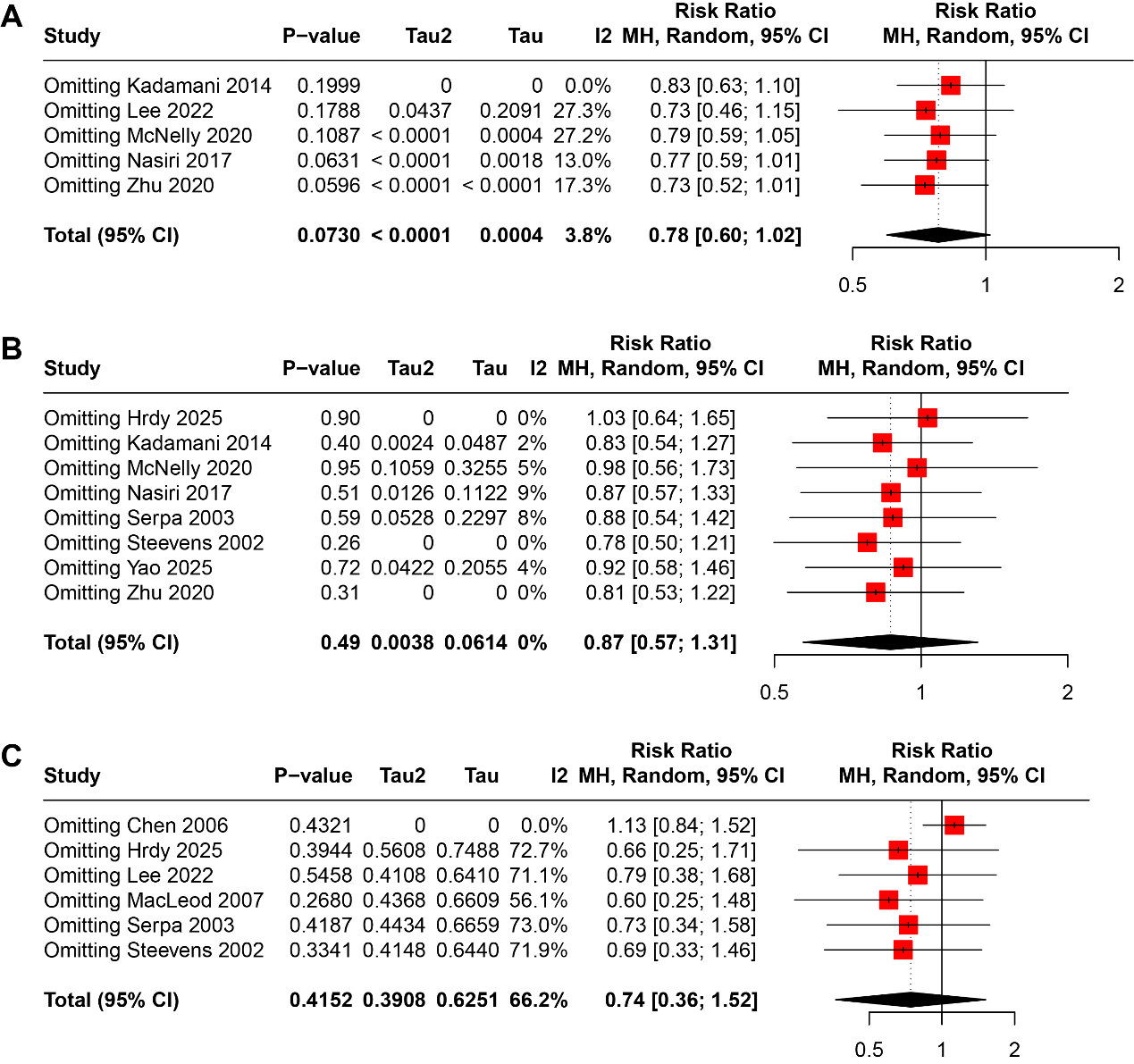


Figure 6: Sensitivity analysis for (A) constipation, (B) gastric retention, (C) aspiration pneumonia


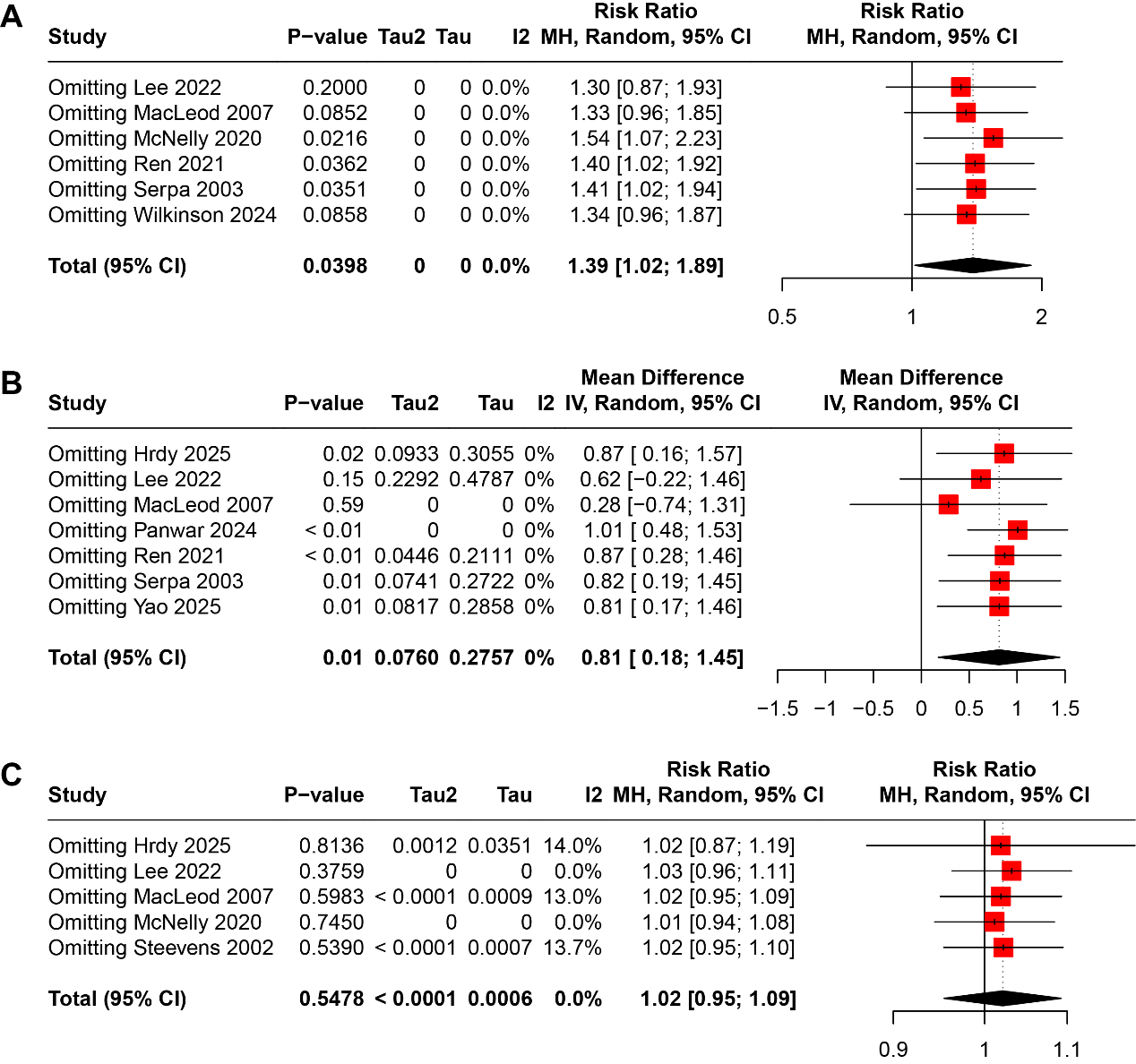


Figure 7: Sensitivity analysis for (A) ICU mortality, (B) length of stay in ICU, (C) achievement of nutritional goal


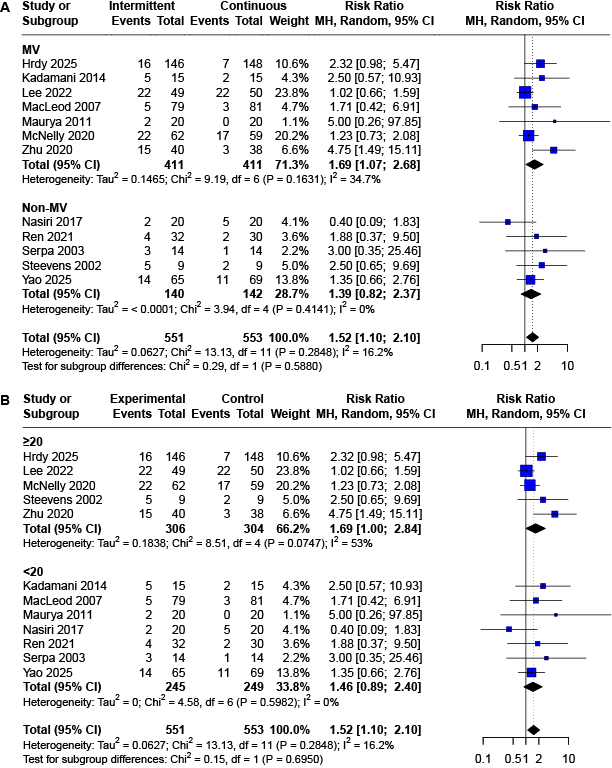


Figure 8: Subgroup analysis for diarrhea, stratified by (A) MV and non-MV subgroup, (B) APACHE II ≥20 and < 20 subgroup


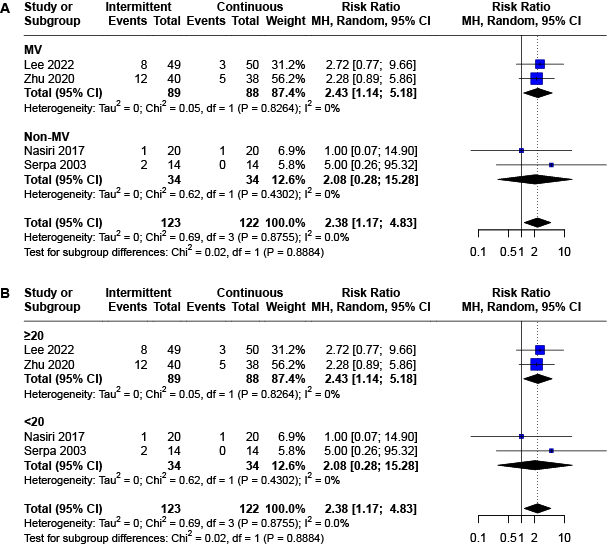


Figure 9: Subgroup analysis for abdominal distension, stratified by (A) MV and non-MV subgroup, (B) APACHE II ≥20 and < 20 subgroup


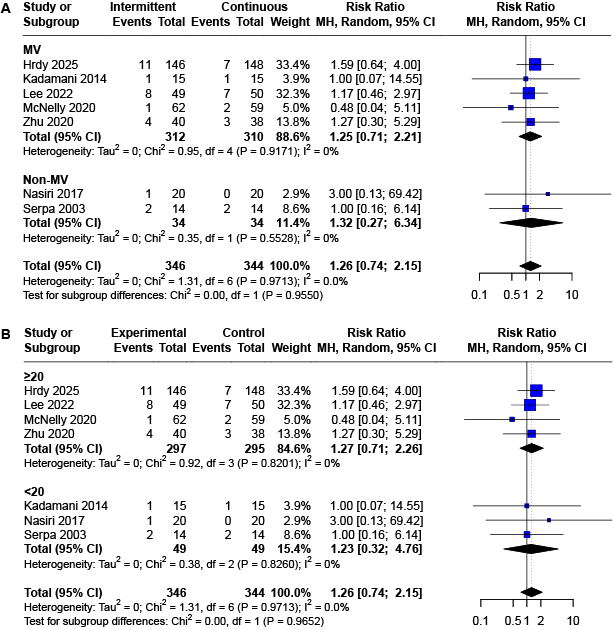


Figure 10: Subgroup analysis for vomiting, stratified by (A) MV and non-MV subgroup, (B) APACHE II ≥20 and < 20 subgroup


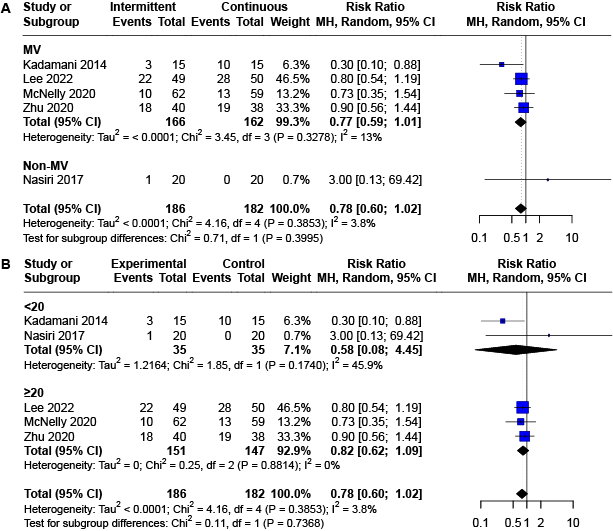


Figure 11: Subgroup analysis for constipation, stratified by (A) MV and non-MV subgroup, (B) APACHE II ≥20 and < 20 subgroup


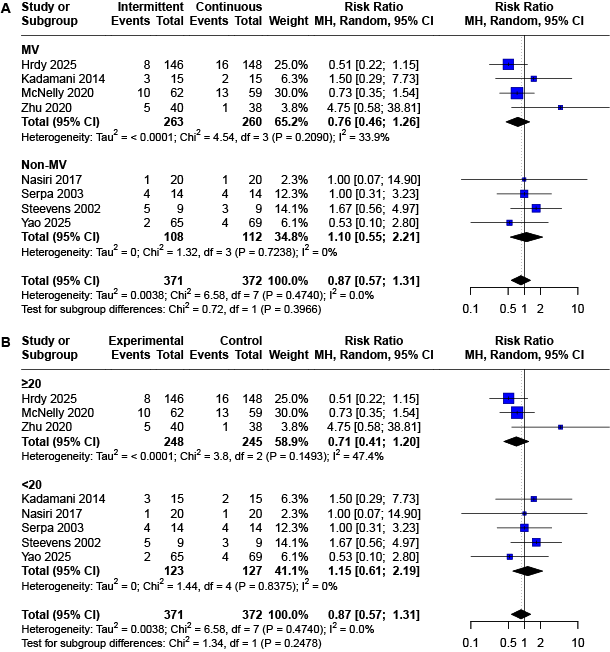


Figure 12: Subgroup analysis for gastric retention, stratified by (A) MV and non-MV subgroup, (B) APACHE II ≥20 and < 20 subgroup


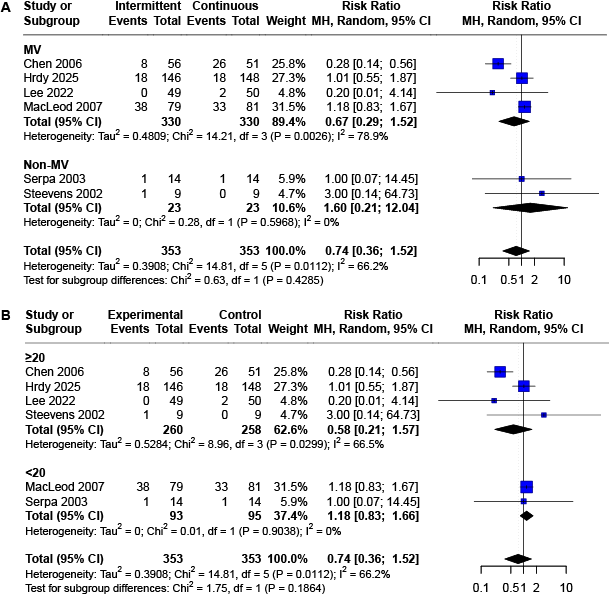


Figure 13: Subgroup analysis for aspiration pneumonia, stratified by (A) MV and non-MV subgroup, (B) APACHE II ≥20 and < 20 subgroup


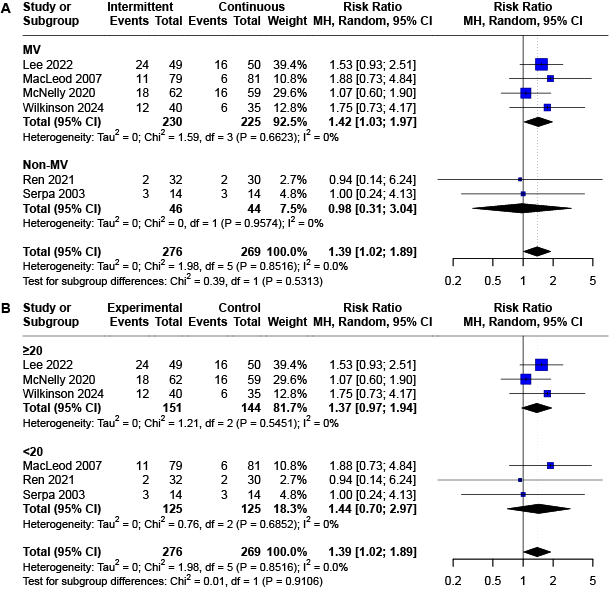


Figure 14: Subgroup analysis for ICU mortality, stratified by (A) MV and non-MV subgroup, (B) APACHE II ≥20 and < 20 subgroup


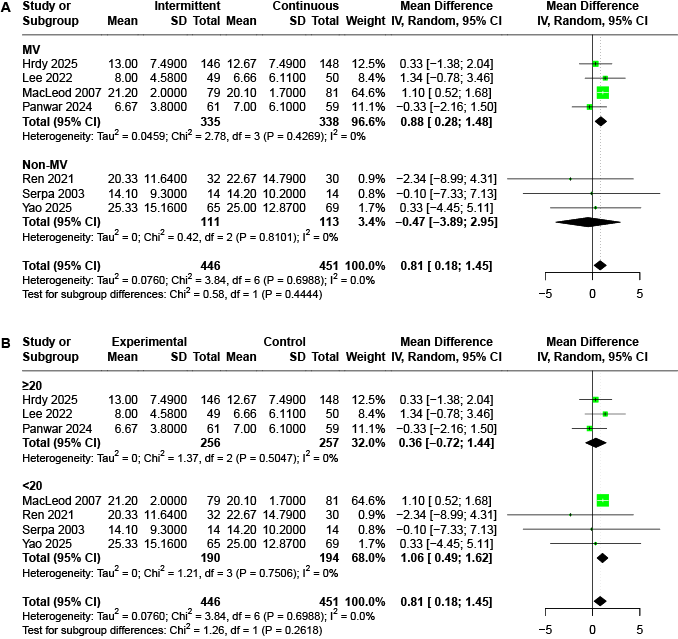


Figure 15: Subgroup analysis for length of stay in ICU, stratified by (A) MV and non-MV subgroup, (B) APACHE II ≥20 and < 20 subgroup


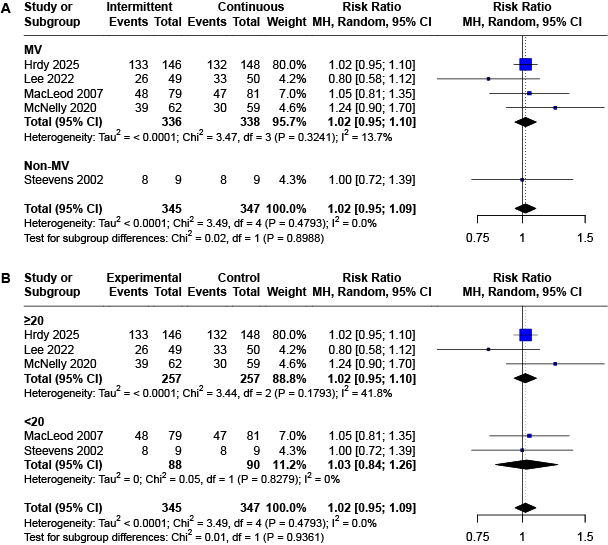


Figure 16: Subgroup analysis for achievement of nutritional goal, stratified by (A) MV and non-MV subgroup, (B) APACHE II ≥20 and < 20 subgroup
